# Case report: PD-1 inhibitor-based treatment strategies in gastric cancer complicated by bone marrow metastasis and disseminated intravascular coagulation: A report of two cases

**DOI:** 10.3389/fonc.2023.1019702

**Published:** 2023-02-22

**Authors:** Ren-Ze Huang, Nuo Chen, Yan Hu, Wan-Ming Hu, Feng-Hua Wang, Dong-Liang Chen

**Affiliations:** ^1^ Department of Medical Oncology, State Key Laboratory of Oncology in South China, Collaborative Innovation Center for Cancer Medicine, Sun Yat-sen University Cancer Center, Guangzhou, China; ^2^ Department of Pathology, State Key Laboratory of Oncology in South China, Collaborative Innovation Center for Cancer Medicine, Sun Yat-sen University Cancer Center, Guangzhou, China

**Keywords:** gastric cancer, bone metastasis, disseminated intravascular coagulation, programmed death protein 1 inhibitors, immunotherapy

## Abstract

**Introduction:**

Gastric cancer (GC) complicated by bone marrow metastasis (BMM) and disseminated intravascular coagulation (DIC) represents poor prognosis and most of these patients would die in a few months. Active treatment strategies such as chemotherapy are effective in restoring coagulation function and prolonging patients’ survival time. Immunotherapy including programmed death protein 1 (PD-1) or programmed death protein ligand 1 (PD-L1) inhibitors has emerged as a first-line treatment of gastric cancer. However, the efficacy of PD-1 inhibitor-based treatment strategies in these patients remains unknown.

**Case description:**

Herein, we presented two cases of advanced gastric cancer (AGC) complicated by BMM and DIC, in which two patients received chemotherapy and PD-1 inhibitor as the first-line treatment. Both of them achieved a partial response after treatment, and the coagulation function was restored. The patient who discontinued the PD-1 inhibitor after 6 months experienced DIC relapse, whereas the other patient who maintained the PD-1 inhibitor treatment cycle remained responsive after 10 months.

**Conclusions:**

We speculate that PD-1 inhibitor-based treatment strategies are effective and safe in prolonging survival against gastric cancer with BMM and DIC, and the coagulation function is well controlled by the treatment with a combination of immunotherapy and chemotherapy.

## Introduction

Gastric cancer (GC) is the fifth most common cancer and the fourth leading cause of cancer-related deaths worldwide, and remains a major health problem (1). In China, although the incidence and mortality of gastric cancer are decreasing annually, the numbers of new cases and deaths are still more than 390,000 and 280,000, respectively (1, 2).

Disseminated intravascular coagulation (DIC) is a severe syndrome, characterized by the pathological intravascular activation of coagulation of blood clotting factors, and usually results in organ dysfunction. Cancer-associated DIC is one of the complications of tumors, and it has been reported in several types of solid tumors, including gastric cancer (3, 4). Bone marrow metastasis (BMM) might be one reason for hematological abnormalities such as DIC and anemia, whereas less than 10% of patients with advanced gastric cancer (AGC) have bone metastasis (5, 6). The prognosis for patients with AGC complicated by DIC remains poor, with the median overall survival (OS) of patients without treatment being less than 3 months (4). Active treatment has been shown to benefit these patients in previous case reports (3, 4, 7–11). However, due to the limited studies of the treatment of AGC complicated by BMM and DIC, the best strategies remain unknown.

Recently, immune checkpoint inhibitors (ICIs), especially inhibitors of programmed death protein 1 (PD-1) and programmed death protein ligand 1 (PD-L1), have emerged as first-line treatment strategies in advanced gastric cancer (12). Many clinical trials have demonstrated that PD-1/PD-L1 inhibitors show impressive clinical efficacy (13–15). The safety profile of ICIs in patients with AGC has been acceptable, as previously reported. However, the efficacy and safety of PD-1 inhibitor-based treatment strategy in AGC complicated by BMM and DIC remain unknown.

As far as we know, the current study is the first to report the application of PD-1 inhibitors in AGC patients having BMM and DIC complications. Two patients were included, and both achieved partial response after the application of ICIs. However, the patient who discontinued the PD-1 inhibitor after 6 months experienced DIC relapse, whereas the other patient who maintained the PD-1 inhibitor treatment cycle remained responsive after 10 months. We observed that active antitumor treatment strategies contributed to restoring coagulation function, especially PD-1 inhibitor-based treatment strategies that are effective in prolonging survival time for cancer patients having DIC complications.

## Case report

### Case 1

A 65-year-old woman was admitted to our hospital on 16 March 2022, with epigastric pain for 2 months. Physical examination showed otherwise normal results, without bruise, bleeding, or other coagulation disorder symptoms. Gastric endoscopy showed hyperplasia, protuberance of cardia mucosa, and a diffuse thickened gastric wall, with a biopsy result that revealed a poorly differentiated gastric adenocarcinoma, classified into diffuse type according to Lauren’s criteria. The results of immunohistochemical staining of gastric neoplasm specimens were negative for HER2, and the Combined Positive Score (CPS) of PD-L1 was 3. Abdominal computer tomography (CT) scanning showed a thickened gastric wall accompanied by multiple metastases including lung, bone, peritoneum, mesentery, and retroperitoneal lymph nodes. A marrow biopsy found that the malignant cell infiltrated the bone marrow. Additionally, results of next-generation sequencing (NGS) revealed microsatellite instability with a high tumor mutation burden (TMB-H) of 11.52 mutations per megabase (muts/Mb).

The blood count test showed platelet (PLT) counts of 72.0 × 10^9^/L. Blood coagulation tests revealed prolonged prothrombin time [PT of 20.3 s; the international normalized ratio (INR) is 1.80], reduced fibrinogen level (Fbg of 0.66 g/L), elevated D-dimer level (39.07 μg/mL), and fibrin degradation product level (FDP of 105.7 μg/mL). Her DIC score was 7 points, according to the recommendation of the International Society on Thrombosis and Haemostasis (ISTH) committee (16). After excluding severe infectious diseases, trauma, or other reasons for DIC, the patient was finally diagnosed with advanced gastric adenocarcinoma accompanied by multiple metastases and DIC complications.

A regimen consisting of modified docetaxel combined with cisplatin, 5-fluorouracil (5-FU), and sintilimab (mDCF plus sintilimab) was initiated. The treatment strategy started on the third hospital day. Best supportive care was provided to the patient until the coagulation function was restored, including a transfusion of fresh frozen plasma, fibrinogen, and heparin. On the 12th day (27 March), the coagulation function was improved, with the platelet count of 274 × 10^9^/L, PT of 11.4 s, INR of 0.99, D-dimer level of 13.96 μg/mL, and FDP level of 21.3 μg/mL. The patient was discharged on the 12th hospital day. The patient received chemotherapy and immunotherapy regularly at our hospital.

**Figure 1:**
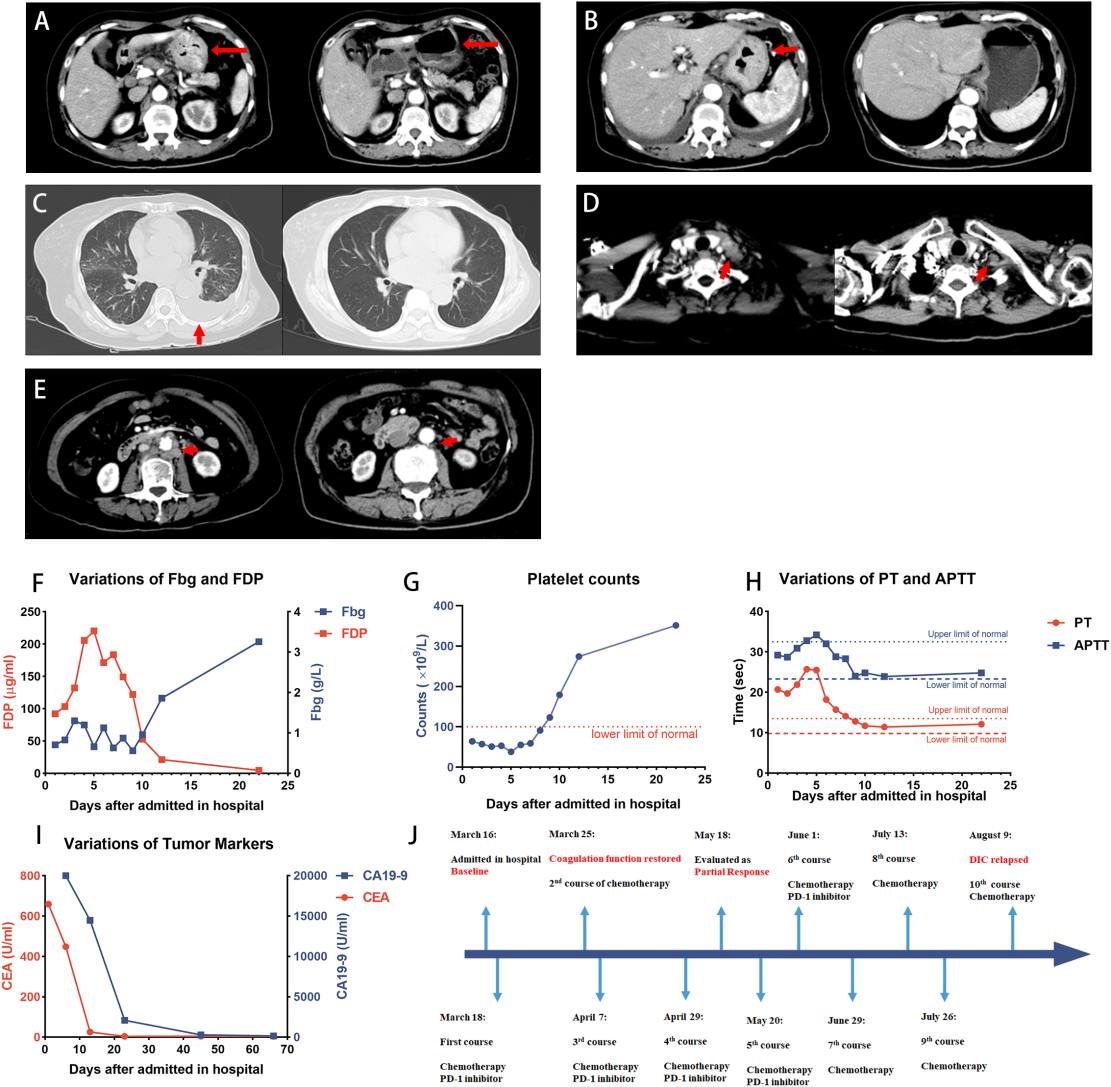
Case 1 patient achieved PR after the treatment. **(A–E)** CT images showing the patient’s baseline disease (left panel) and response to treatment (right panel). **(A, B)** Diffuse thickened gastric wall significantly thinned after the treatment. **(C)** Pleural effusion indicated the left lung metastasis, and it was absorbed after the treatment. **(D)** Virchow’s nodes were slightly reduced in size. **(E)** Retroperitoneal lymph nodes shrunk after the treatment. **(F–H)** Dynamic monitoring of Fbg level, FDP, platelet counts, PT, and APTT, suggested that the coagulation function was restored. **(I)** Dynamic monitoring of tumor markers during the treatment. **(J)** Timeline of the treatment. The patient discontinued PD-1 inhibitors on 29 June and received chemotherapy only, and DIC relapsed after 2 months. PR, partial response; Fbg, fibrinogen; FDP, fibrin degradation product; PT, prothrombin time; APTT, activated partial thromboplastin time.

After three courses of treatment, the patient achieved partial response (**Figure 1**), according to the Response Evaluation Criteria in Solid Tumors version 1.1 (RECIST) (17). A CT scan performed on 18 May revealed that the lesions in the stomach, peritoneum, and metastases of lymph nodes were reduced, whereas the lesion in the bone was stable. A significant decrease in tumor markers and improvement in coagulation tests indicated that gastric cancer was well controlled. However, due to the heavy economic burden, the patient decided to discontinue immunotherapy and received chemotherapy only on 29 June. After two courses of chemotherapy were given to the patient, laboratory tests showed a PLT count of 112 × 10^9^/L, PT of 16.4 s, INR of 1.43, Fbg level of 0.36 g/L, D-dimer level of 23.42 μg/mL, and FDP of 33.1 μg/mL, suggesting that the DIC relapsed. The latest chemotherapy was given on 10 August, and the patient discontinued the treatment in our hospital. The progression-free survival was 6 months. No serious treatment-related adverse events were reported during the treatment.

### Case 2

A 43-year-old man presented with epigastric pain and low back pain for 3 months and was admitted to our hospital on 28 January 2022. Endoscopy and biopsy identified a poorly differentiated gastric adenocarcinoma, classified into diffuse type. Immunohistochemical staining results were positive for HER2, the CPS of PD-L1 was 1, and microsatellite instability was detected. A CT scan revealed a thickened gastric wall, multiple metastases of lymph nodes, and multiple lesions in thoracic and lumbar vertebrae, suggesting that the patient had gastric cancer complicated by bone metastasis. Anemia was detected according to the blood count on 8 February, which showed a red blood cell (RBC) count of 2.92 × 10^12^/L, hemoglobin (HGB) of 78.0 g/L, and a platelet count of 97.0 × 10^9^/L. The coagulation test showed prolonged PT of 20.3 s, INR of 1.80, an elevated D-dimer level of 73.56 μg/mL, and an FDP level of 206.4 μg/mL). He was diagnosed with non-overt DIC because his score of 4 was less than the cutoff of 5 (16). On 17 February, another test revealed an RBC count of 2.54 × 10^12^/L, HGB of 67.0 g/L, platelet count of 57.0 × 10^9^/L, PT of 13.5 s, INR of 1.18, D-dimer level of 64.41 μg/mL, and FDP of 192.9 μg/mL.

After a diagnosis of advanced gastric carcinoma with multiple metastases, his therapy was initiated on 17 February, with a regimen of XELOX combined with trastuzumab and an immune checkpoint inhibitor, sintilimab. Supportive care of low molecular weight heparin (LMWH) was given to prevent venous thromboembolism (VTE) and DIC until the platelet counts and hemoglobin returned to the normal range. Zoledronic acid was also given because of bone metastases. After the first course of treatment, the patient’s coagulation function was restored, and the hematologic profile improved.

**Figure 2:**
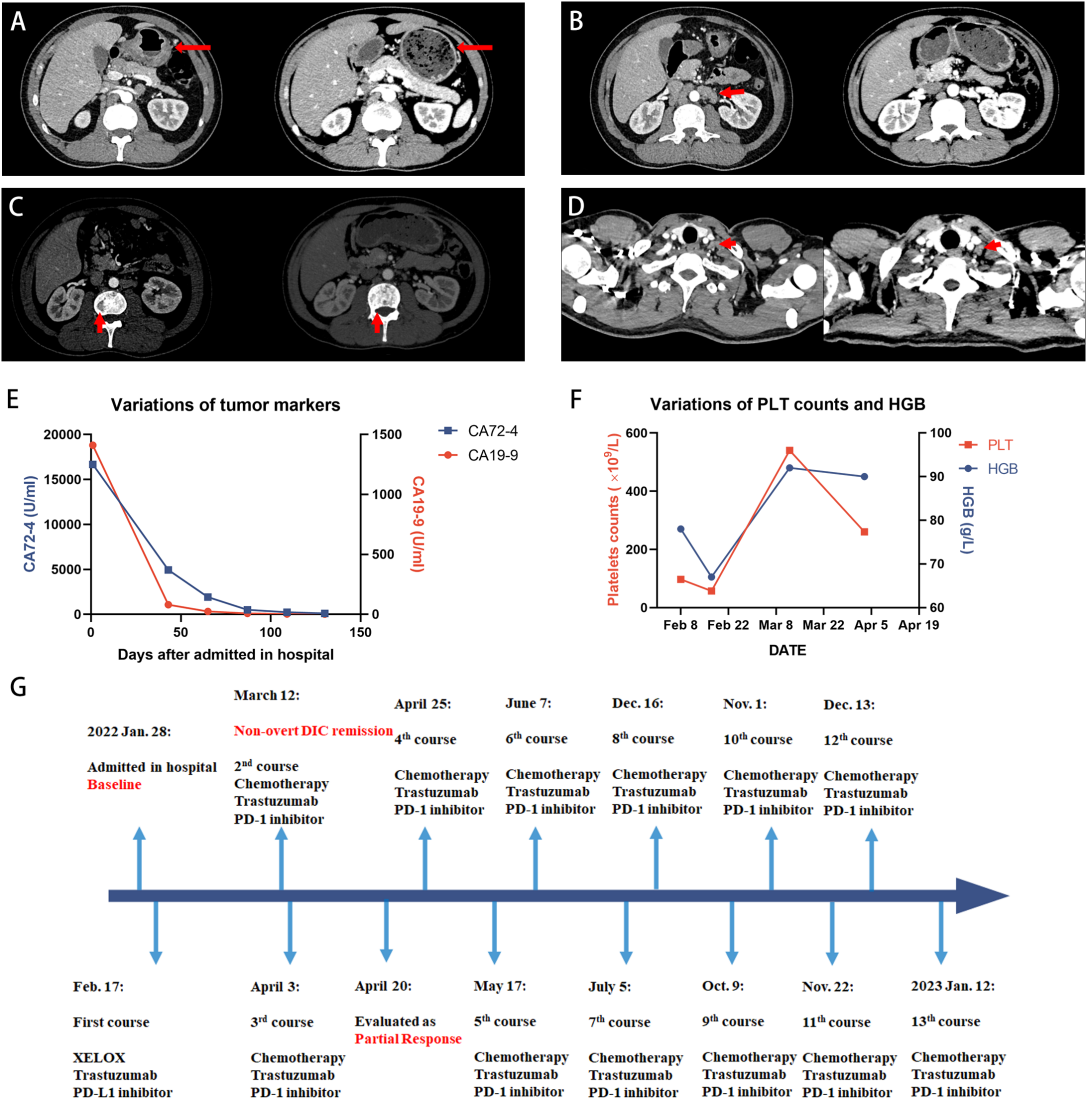
Case 2 patient achieved PR after the treatment. **(A-D)** The outcomes of CT scans at baseline (left panel) and 2 months after the treatment (right panel). **(A)** Thickened gastric wall thinned after the treatment. **(B)** Retroperitoneal lymph nodes significantly shrunk compared with the baseline. **(C)** The uneven density of the lumbar vertebrae was kept stable during the treatment. **(D)** Virchow’s lymph nodes are slightly reduced in size. **(E)** The decline in the tumor marker level suggested that the treatment was effective. **(F)** Anemia remission and coagulation function were restored according to the dynamic monitoring of PLT counts and HGB level. PLT counts temporarily increased in the over-normal range on 8 March but finally returned to the normal range. **(G)** Timeline of the treatment. PLT, platelet; HGB, hemoglobin.

The patient achieved partial response after 2 months (**Figure 2**). On 19 April, CT images revealed a significant reduction in thickened gastric wall and metastases of lymph nodes, whereas lesions in bone were kept stable. The level of tumor markers also decreased significantly. Now he has finished 13 courses of treatment and continues to receive treatment in our hospital, with progression-free survival of 10 months. No serious treatment-related adverse events were reported during the treatment.

## Discussion

We herein present two cases of PD-1 inhibitor-based treatment strategies in AGC with BMM and DIC. Although the patient of case 2 had not yet been diagnosed with DIC, he was classified into a subclinical type of cancer-related DIC because we believed that the tendency to progress to DIC is high (18). Both patients achieved a partial response after the treatment, and the coagulation function was restored.

Gastric cancers have a high probability of metastasizing to the liver and lung, but only less than 10% of the cancers metastasize to the bone (5). Malignant cells infiltrating the bone marrow space might result in anemia, thrombocytopenia, DIC, or other hematologic disorders. The prognosis for patients with AGC complicated by DIC remains poor, and the median OS of these patients without treatment was less than 3 months (4). Mainly reported in the form of case reports, the clinical characteristics of AGC with bone marrow metastasis and DIC remain unclear, and a standard treatment needs to be established.

DIC is an acquired syndrome originating from infectious or non-infectious diseases. One of the most frequent factors that promote DIC is sepsis. However, neither fever nor other symptoms of sepsis were presented in our two cases, suggesting that coagulation disorders were probably triggered by gastric cancer. Cancer-induced DIC is usually related to high expression of tissue factors, which can activate Factor XII and generate thrombin (19). DIC occurs due to the failure of controlling thrombin generation. Overexpression of fibrinolytic proteins may contribute to a hyperfibrinolytic condition (20). Cytokines may play a significant role in the pathogenesis of cancer-related DIC. Due to lack of data from high-quality research, the pathophysiology of cancer-related DIC remains unclear and needs to be further investigated. Treatment of primary diseases may relieve this severe syndrome.

Necrosis of malignant cells caused by chemotherapy may disrupt endothelial cells, aggravating the progression of DIC. Hematological abnormalities and poor performance status also raise the question on the tolerability and efficacy of chemotherapy. However, DIC management is promptly recognized, and appropriate management of the underlying disease is the main principle of treatment, according to the guidance of ISTH (16, 18). Previous retrospective studies have reported that chemotherapy for AGC with DIC has greater survival benefits than the best supportive care (9, 21). Chemotherapy regimens based on platinum, docetaxel, or fluoropyrimidine were most often used in the treatment (3, 4, 7–11). A study demonstrated that the chemotherapy regimen of 5-FU plus docetaxel was associated with prolonged survival after comparing different clinical treatments (4). However, the best treatment strategy remains under investigation.

Recently, immunotherapy has demonstrated impressive clinical efficacy and safety in the treatment of AGC. Immune checkpoint inhibitors (ICIs), such as PD-1/PD-L1 blockade, are the most commonly used agents in AGC. Nivolumab provided superior OS versus placebo in AGC patients regardless of the expression level of PD-L1, and nivolumab plus chemotherapy demonstrated significant improvement in OS versus chemotherapy, according to ATTRACTION-2 and CheckMate 649 studies (13, 15). Sintilimab plus XELOX performed better than placebo plus XELOX in the treatment of AGC, as reported by an ORIENT-16 study (14). The addition of trastuzumab, an antibody inhibitor of human epidermal growth factor receptor 2 (HER-2), to pembrolizumab and chemotherapy in the treatment of HER-2-positive gastric cancer also markedly reduced the tumor size and significantly improved the objective response rate (22). The safety profile of the addition of ICIs was consistent with the known safety profiles, suggesting that immunotherapy is safe (13–15, 22, 23). The main adverse events include dermatologic, gastrointestinal, and endocrine toxicities, whereas the hematological toxicity of ICIs is rare (24). The strategy of immunotherapy plus chemotherapy may thus have a potential for patients with cancers complicated by bone marrow metastasis and DIC.

We speculate that immunotherapy-based strategies would provide great efficacy in the treatment of gastric cancer-related DIC. After reviewing previous literature in PubMed and EMBASE, only two case reports were identified, which described the significant efficacy of atezolizumab and pembrolizumab in the treatment of urothelial carcinoma and bone marrow metastatic melanoma, respectively (25, 26). In our cases, two patients achieved partial response and their coagulation function was restored after treatment. However, the condition of the case 1 patient who discontinued immunotherapy on 29 June then received two courses of chemotherapy, worsened with DIC, and relapsed 2 months later, whereas case 2 patient is still receiving chemotherapy, targeted therapy, and immunotherapy in our hospital, with indicators of DIC still kept in the normal range. Different clinical outcomes of the case 1 patient with or without the PD-1 inhibitor may indicate that the combination of chemotherapy and immunotherapy would be better than chemotherapy alone. Unfortunately, few cases of the PD-1-based treatment strategy in cancer-related DIC are reported here and the findings need to be confirmed by larger cohorts of patients.

Our study is further constrained by the limitation of a case report. We did not set up a control group of AGC complicated by BMM and DIC which only received chemotherapy. Due to the limited number of these patients, it is difficult for us to set up such a control group. Therefore, it can hardly confirm the efficacy of PD-1 inhibitors alone in the treatment of these patients. Since cancer cells could express procoagulant molecules and cytokines, or cause microangiopathy to induce DIC, the treatment of primary cancer may be an effective strategy (19). We have a reason to speculate that immunotherapy would yield great efficacy in the treatment of cancer-related DIC. However, whether immunotherapy can be effective in AGC complicated by BMM and DIC or in minimizing adverse events still needs to be studied. Our conclusion still needs further validation in prospective clinical trials and basic experiments.

In summary, we presented two cases of PD-1 inhibitor-based treatment strategies in prolonging survival for patients with AGC with BMM and DIC whose prognosis is poor, and it could be considered as an initial treatment strategy for AGC with BMM and DIC.

## Data availability statement

The original contributions presented in the study are included in the article. Further inquiries can be directed to the corresponding authors.

## Ethics statement

The studies involving human participants were reviewed and approved by the Institutional Research Ethics Committee of Sun Yat-sen University Cancer Center. The patients/participants provided their written informed consent to participate in this study.

## Author contributions

R-ZH, NC, and YH analyzed the patient data and drafted the manuscript. W-MH provided significant contributions to the analysis of pathological data. F-HW and D-LC designed the case report and revised the manuscript. All authors contributed to the article and approved the submitted version.
